# Screening Strategies Among High‐Risk Populations for Anal Cancer (ACCESS Study) Protocol

**DOI:** 10.1002/cpz1.70518

**Published:** 2026-11-11

**Authors:** Raju Subbiah, Sohan Rodney Bangera, Nadi N. Kaonga, Mary Grace Tadros, Isabella Rosa‐Cuhna, Patricia Paule Jeudin, Kadera Jana Lymon, Nichole A. Ciers‐Davis, Annabel Reyes, Sakthivel Govindaraj, Monica T. Garcia‐Buitrago, C. Christina Mehta, Johnny H. Galli, JoNell E. Potter, Michaela Peters, Alicia K. Smith, Chris Ibegbu, Marina Mosanjac, Vijayakumar Velu, Canhua Xiao, Lisa Flowers

**Affiliations:** ^1^ Division of Microbiology and Immunology, Emory National Biomedical Research Center (ENBRC), Emory Vaccine Center Emory University Atlanta GA USA; ^2^ Department of Pathology and Laboratory Medicine, School of Medicine Emory University Atlanta GA USA; ^3^ Department of Gynecology and Obstetrics, School of Medicine Emory University Atlanta GA USA; ^4^ Department of Medicine, Division of Infectious Diseases, Miller School of Medicine University of Miami Miami FL USA; ^5^ Department of Obstetrics, Gynecology and Reproductive Sciences University of Miami, Leonard M. Miller School of Medicine Miami FL USA; ^6^ Department of Pathology and Laboratory Medicine, Miller School of Medicine University of Miami Miami FL USA; ^7^ Department of Medicine, School of Medicine Emory University Atlanta GA USA; ^8^ Department of Biostatistics and Bioinformatics, School of Medicine Emory University Atlanta GA USA; ^9^ Nell Hodgson Woodruff School of Nursing Emory University Atlanta GA USA; ^10^ Grady Infectious Disease Program Grady Health System Atlanta GA USA; ^11^ Raju Subbiah and Sohan Rodney Bangera contributed equally to this work and share first authorship

**Keywords:** aHSIL, anal cancer, HIV, human papillomavirus, immune profiling

## Abstract

**Background**: Anal cancer is a human papillomavirus (HPV)‐associated malignancy that disproportionately affects high‐risk populations, including people living with HIV, men who have sex with men, and women with a history of high‐grade lower genital tract neoplasia. Persistent infection with high‐risk human papillomavirus (hrHPV) is the primary cause of anal high‐grade squamous intraepithelial lesions (aHSIL), the precursor to anal cancer. Current screening approaches, including anal cytology and high‐resolution anoscopy (HRA), have important limitations related to diagnostic performance, cost, accessibility, and the availability of trained providers. There is a critical need for improved screening strategies and biomarkers capable of identifying individuals at greatest risk of persistent disease and progression.

**Methods**: The screening strategies among high‐risk populations for anal cancer (ACCESS) study is a prospective, multisite, longitudinal cohort study that will enroll 500 high‐risk adults from Emory University and the University of Miami. Participants will undergo annual screening for up to 2 years using anal cytology, hrHPV genotyping, CINtec®PLUS dual staining, HRA, and biopsy when indicated. Anal swabs, biopsies, and optional peripheral blood samples will be collected for molecular and immunologic analyses. DNA methylation markers, inflammatory biomarkers, immune profiling by multicolor flow cytometry, and single‐cell RNA sequencing will be evaluated to assess their association with aHSIL prevalence and clearance. Diagnostic performance measures, multivariable regression analyses, and biomarker‐based predictive models will be used to evaluate study outcomes.

**Discussion**: The ACCESS study will evaluate the performance of emerging screening biomarkers and characterize molecular and immune pathways associated with anal carcinogenesis in diverse high‐risk populations. By integrating clinical, epigenetic, immunologic, and environmental data, this study aims to improve risk stratification, identify biomarkers associated with persistent disease, and inform the development of more effective and targeted anal cancer prevention strategies. © 2026 The Author(s). *Current Protocols* published by Wiley Periodicals LLC.

**Abbreviations**
ACCESS: Screening Strategies Among High‐Risk Populations for Anal Cancer
ACTB: Beta‐actin
ADI: Area Deprivation Index
aHSIL: Anal High‐Grade Squamous Intraepithelial Lesion
AIN: Anal Intraepithelial Neoplasia
ASC‐H: Atypical Squamous Cells, Cannot Exclude HSIL
ASCUS: Atypical Squamous Cells of Undetermined Significance
CINtec®PLUS: p16/Ki‐67 Dual‐Stain Cytology Assay
DARE: Digital Anorectal Examination
EMR: Electronic Medical Record
HIV: Human Immunodeficiency Virus
HRA: High‐Resolution Anoscopy
HPV: Human Papillomavirus
hrHPV: High‐Risk Human Papillomavirus
HSIL: High‐Grade Squamous Intraepithelial Lesion
IRB: Institutional Review Board
LGTN: Lower Genital Tract Neoplasia
MSM: Men Who Have Sex with Men
PBMC: Peripheral Blood Mononuclear Cell
PCR: Polymerase Chain Reaction
qMSP: Quantitative Methylation‐Specific PCR
REDCap: Research Electronic Data Capture
scRNA‐seq: Single‐Cell RNA Sequencing
SEER: Surveillance, Epidemiology, and End Results

## Background and rationale {6a}

Nearly all anal cancers are caused by persistent infection with high‐risk human papillomavirus (hrHPV), and their development is typically preceded by anal high‐grade squamous intraepithelial lesions (aHSIL) (Alemany et al., 2015). According to the National Cancer Institute's Surveillance, Epidemiology, and End Results (SEER) Program, an estimated 11,270 new anal cancer cases and 1,700 deaths will occur in the United States in 2026, reflecting a sustained increase in both incidence and mortality over recent decades (SEER, 2026). The incidence and mortality of advanced anal cancer have been increasing in the United States, with the greatest burden of disease and mortality in individuals living with HIV (Deshmukh et al., 2020). Men who have sex with men (MSM), and women with vulvar and cervical HSIL, or cancer, have the greatest risk of anal disease. Within these groups, Black MSM have significantly higher rates of anal dysplasia and HPV infection than their White counterparts (Walsh et al., 2015). As a result, screening for aHSIL, particularly among populations at risk for hrHPV, may be crucial to mitigating these disparities. Such a practice is supported by findings from the NCI‐sponsored Anal Cancer/HSIL Outcomes Research (ANCHOR) study, which demonstrates that treating aHSIL reduces progression to anal cancer (Palefsky et al., 2022). However, earlier reports noted that screening strategies such as anal cytology and high‐resolution anoscopy (HRA) with biopsy lacked rigorous evidence‐based support (Stewart et al., 2018). Studies have found that anal cytology alone is limited and may not accurately determine lesion grade compared with HRA, which is resource‐intensive, costly, and available through a limited number of trained providers (Gaisa et al., 2014; Roberts et al., 2017). Our preliminary data, along with limited evidence from the literature, suggest that combining screening strategies has the potential to more efficiently triage patients for HRA (Heard et al., 2015; Wentzensen et al., 2014). We propose a large multisite study, focusing on high‐risk populations, to examine the combined effects of multiple less costly and cumbersome screening strategies, with a goal of informing clinical guidelines.

Not all aHSIL will progress to invasive cancer, so the current recommendation to treat all detected aHSIL, regardless of their malignant potential, can result in significant overtreatment (Machalek et al., 2012). Therefore, there is also an urgent need for biomarkers to distinguish aHSIL that will persist and have the highest oncogenic potential. Aberrant DNA methylation of specific host genes (e.g., *ZNF582, ASCL1*) has been associated with persistent hrHPV infection, aHSIL, and HPV‐related cancers (Hernandez et al., 2012; van der Zee et al., 2019). However, studies of these promising biomarkers are not conclusive, due to limitations of small sample sizes, single high‐risk groups, and cross‐sectional designs (Hernandez et al., 2012; Lorincz et al., 2017; van der Zee et al., 2019; Zhang et al., 2005). Longitudinal studies in diverse populations with varying risk factors are needed to capture the full spectrum of anal carcinogenesis. Such studies would provide robust evidence to identify individuals with persistent aHSIL who are at the greatest risk of progression to malignancy (Lee et al., 2018; Tinmouth et al., 2016). Furthermore, because an individual's local immune microenvironment and inflammatory response play a crucial role in anal neoplastic progression, elucidating immunologic profiles could lead to the development of new immunotherapeutic approaches and, eventually, better outcomes (Bojko et al., 2018; Dean & Fenton, 2010; Herd et al., 2018; Korn et al., 2022; Liu et al., 2018; Maness & Thompson, 2019; Stenzel et al., 2021; WHO, 2010; Yaghoobi et al., 2011). We will examine whether environmental covariates play a pivotal role in aHSIL prevalence and clearance by recruiting and following 500 high‑risk men and women over a 2‑year period from the catchment areas of Emory University and the University of Miami. This approach will provide critical insights into disease progression and inform targeted prevention and intervention strategies for anal cancer. To address these gaps, this study is designed around three specific aims focusing on screening performance and molecular biomarkers in aHSIL progression and informing targeted prevention and intervention strategies for anal cancer.

## Objectives {7}

Aim 1: Compare sensitivity and specificity of emerging screening strategies (CINtec®PLUS, hrHPV, and anal cytology) for detection of aHSIL (HRA biopsy‐proven) in a high‐risk population.

Hypothesis 1: Emerging screening strategies will have higher sensitivity and specificity for detecting aHSIL compared to anal cytology alone.

Aim 2: Assess the association between anal DNA methylation markers (*ZNF582, ASCL1, ZIC1*, *SST, LHX8, WDR17, and ST6GALNAC5*) and anal immune/inflammatory markers with aHSIL (prevalence and clearance) among high‐risk groups.

Hypothesis 2a: DNA methylation markers are associated with aHSIL.

Hypothesis 2b: Immune profiles and inflammatory markers are associated with aHSIL.

## Trial design {8}

The ACCESS study is strategically designed as a multisite, longitudinal cohort. Recruitment is across two geographically distinct academic medical centers, increasing the generalizability of results. The 2‐year follow‐up period balances feasibility with biological relevance for observing disease progression or regression. The total duration of the study will be 5 years (Figure 1).

**Figure 1:**
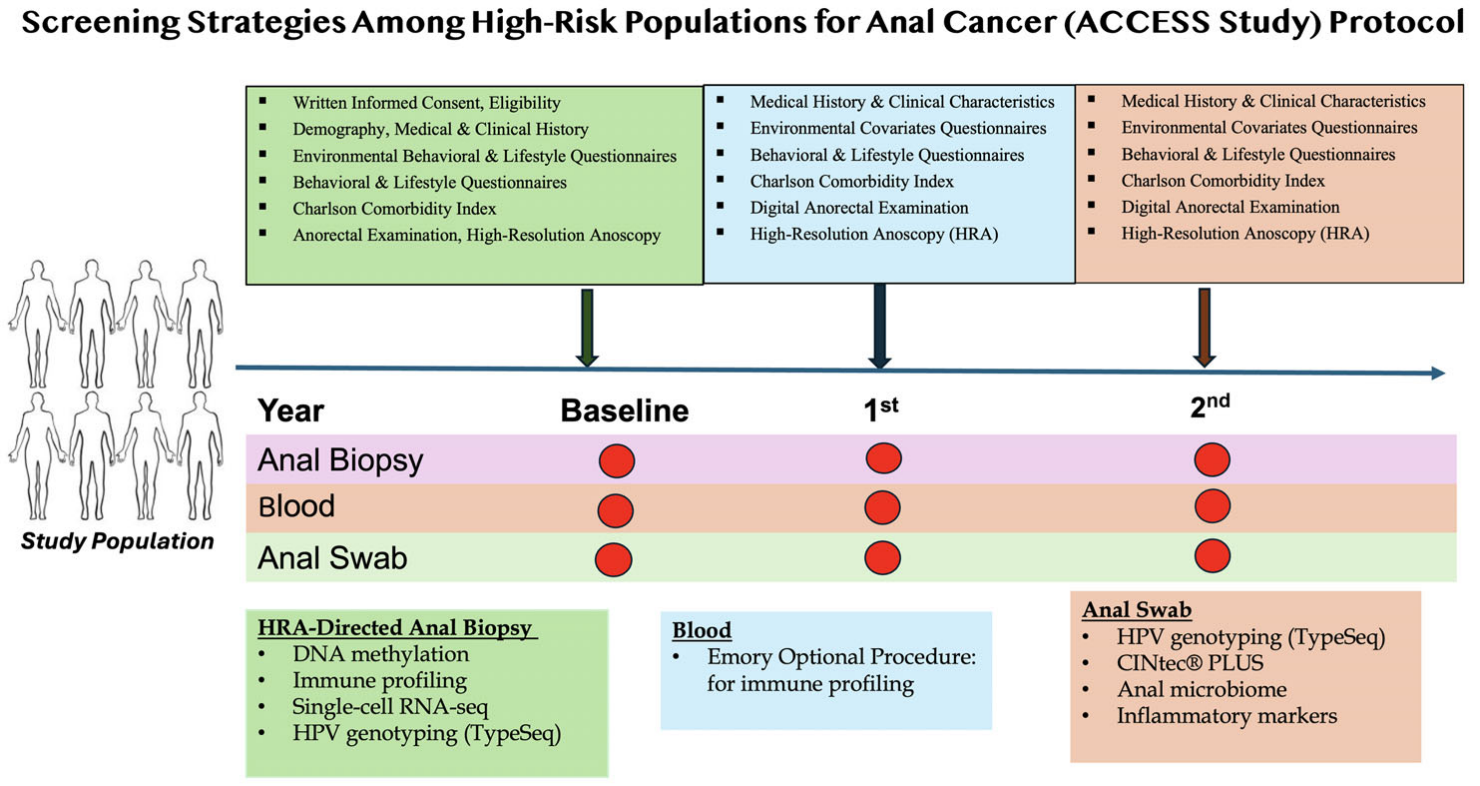
Study timeline for the ACCESS study. Participants are followed at baseline, Year 1, and Year 2 with specified clinical, laboratory, and questionnaire‐based assessments.

## Methods: Participants, interventions, and outcomes

### Study setting {9}

We will recruit 500 study participants, 250 from Emory University, Atlanta, USA clinical sites and 250 from University of Miami, Miami, USA clinical sites. Though the study will be open to eligible individuals receiving care throughout both healthcare systems, most study participants will be recruited from the Grady Memorial Hospital Georgia Cancer Center of Excellence Colposcopy and Anoscopy Clinics and the Ponce de Leon Center Anoscopy Clinic at Emory University and from the Anal Dysplasia clinics at Jackson Memorial Hospital (JMH) and at the Sylvester Comprehensive Cancer Center (SCCC) at the University of Miami during routine scheduled anal cancer screening examinations. These clinics are supervised by Dr Flowers at Emory University and Dr Rosa‐Cunha at both sites for the University of Miami. We will request a waiver of consent for screening potential participants for the study. Inclusion and exclusion criteria will be assessed by reviewing medical records and prescreening potential participants via the phone.

### Eligibility criteria {10}

After Institutional Review Board (IRB) approval and HIPAA authorization, trained research assistants (RA) will screen participating physicians’ clinic schedules for eligible patients. The RA will meet with eligible participants to obtain consent. Inclusion criteria: (1) Individuals with chronic impairment of the immune system and (2) females with known history of high‐grade lower genital tract neoplasia (LGTN), Zubrod Performance Status of 0–2, ≥ 30 years of age, and ≤ 80 years of age. Women with known high‐grade LGTN are women with HPV‐related cervical, vaginal, or vulvar high‐grade dysplasia or cancer. Women not on long‐acting reversible contraception or who have not undergone permanent sterilization will have a urine pregnancy test performed prior to signing the consent.

Exclusion criteria will include patients treated for aHSIL less than 6 months prior to screening, history of anal cancer, and pregnant women. Past medical history of the study participants will be extracted from the electronic medical record (EMR) systems of Emory University and the University of Miami, and from participant interviews. Exclusion criteria include individuals unable to provide consent, minors, pregnant women, prisoners, cognitively impaired individuals, those treated for aHSIL within 6 months, and those with a history of anal cancer. Individuals with a prior diagnosis of anal cancer will be excluded from the study. This exclusion is necessary because prior treatment for anal cancer, including surgery, chemotherapy, radiation therapy, or immunotherapy, may alter tissue architecture, HPV status, immune responses, and biomarker profiles. Excluding these individuals will allow a more accurate evaluation of screening strategies, biomarkers, and immune correlates associated with aHSIL prevalence, persistence, and clearance in populations at risk for anal cancer. In the current study, we do not intend to evaluate the role of economic deprivation.

### Who will take informed consent? {26a}

Trained research assistants and study personnel approved by the IRBs at participating institutions will obtain written informed consent from eligible participants prior to enrollment and before the initiation of any study‐related procedures. During the consenting process at the clinic, to minimize the possibility of coercion or undue influence, research team members conducting consent will clearly explain that participation in the study is completely voluntary and that participation or nonparticipation will not affect the participant or their health care. The informed consent document will also clearly state this: “Patients who are not native English speakers were consented in their primary language to ensure their understanding of the study.” Additionally, all research staff will be trained and undergo IRB‐required clinical research training before contacting participants. After reviewing the informed consent document, potential participants will be provided the time needed to ask any questions they may have and decide if they want to participate in the study. A copy of the signed consent form will be given to the participants, and a copy will be placed in a locked file in a locked office and will be kept separate from any data gathered as part of the study. As part of the consent process, participants will be informed of the $60 compensation for participating in each study visit, with a total of $180 if a participant completes all study visits.

### Additional consent provisions for collection and use of participant data and biological specimens {26b}

Participants will be asked to provide separate optional consent for the storage and future use of peripheral blood specimens, including peripheral blood mononuclear cells (PBMCs) and plasma, for future research related to anal cancer, HPV‐associated disease, and immune responses. Participation in these optional activities is not required for enrollment in the ACCESS study. Participants may not provide consent for storage and future use of specimens while remaining eligible for all primary study procedures.

## Interventions

### Explanation for the choice of comparators {6b}

HRA‐directed biopsy with histologic confirmation serves as the reference standard for aHSIL diagnosis. The diagnostic performance of emerging screening strategies, including anal cytology, hrHPV testing, and CINtec®PLUS dual staining, will be evaluated against biopsy‐confirmed disease status.

### Intervention description {11a}

At each study visit, an HRA‐certified physician will collect anal swabs and HRA‐targeted biopsies for histological confirmation of disease and biomarker assays. Participants will undergo HRA performed by physicians who have completed formal HRA training and experience in the identification and biopsy of anal squamous intraepithelial lesions. Participants with substantial histopathologic confirmation of biopsied lesions will serve as the reference standard for aHSIL diagnosis. Four consecutive samples of exfoliated cells from the anal canal will be collected using polyester swabs. Swabs will be placed in the specified buffers/media according to the sample collection standard operating procedure. The samples will be used as follows: 1) one for anal cytology and CINtec®PLUS, 2) one for hrHPV testing, 3) one for DNA methylation analysis, and 4) one for inflammatory marker analysis. Additionally, an extra sample will be collected for future research if the participant has consented to this optional use.

#### HRA and histology

All participants will receive a digital anorectal examination (DARE) followed by HRA, with directed biopsies of suspicious lesions. The biopsies will be reported in accordance with the terminology, criteria, and recommendations of the Lower Anogenital Squamous Terminology (LAST) project (Darragh et al., 2012). In brief, histology results will be reported as benign (negative for squamous intraepithelial lesion), AIN grades 1–3, or cancer. For the proposed project, histologic findings of aHSIL will be defined as AIN2 (p16 block‐positive), AIN2‐3, and AIN3. Any cases of cancer will be noted. All other cases will be classified as <aHSIL, including benign, condyloma acuminatum, and AIN1.

#### Anal cytology

The collected cells, suspended in the SurePath or ThinPrep liquid‐based medium, will be placed on a slide and microscopically examined by a board‐certified cytopathologist. The sample will then be interpreted using the Bethesda System: negative for intraepithelial lesion or malignancy (NILM), ASCUS, LSIL, ASC‐H, or HSIL. Pathologists interpreting the anal cytology will be blinded to hrHPV testing and biopsy histology.

#### Genotyping of anal hrHPV infection

To identify the infecting HPV genotype, DNA will be extracted from swab samples collected at baseline, Year 1, and Year 2 with the MagNA Pure LC DNA isolation Kit III and an LC MagNA Pure system (Roche Diagnostics GmbH, Mannheim, Germany), followed by HPV typing using the TypeSeq assay, a laboratory‐prepared high‐throughput next‐generation sequencing assay developed by NCI will be performed as described previously (Wagner et al., 2019). The assay uses three sequential PCR steps resulting in barcoded L1 amplicons that are sequenced and submitted to a bioinformatics pipeline for typing (Wagner et al., 2019). This TypeSeq assay qualitatively detects and identifies 51 HPV genotypes (HPV types 3, 6, 11, 13, 16, 18, 26, 28, 30, 31, 32, 33, 34, 35, 39, 40, 42, 43, 44, 45, 51, 52, 53, 54, 56, 58, 59, 61, 62, 66, 67, 68, 69, 70, 71, 72, 73, 74, 76, 81, 82, 83, 84, 85, 86, 87, 89, 90, 91, 97, and 114), including 14 high‐risk types (16, 18, 31, 33, 35, 39, 45, 51, 52, 56, 58, 59, 66, and 68) (Gillison et al., 2012; Muñoz et al., 2003; Wagner et al., 2019). As needed, sections of the diagnostic archived anal biopsies may be tested as well, as the assay has been validated for formalin‐fixed, paraffin‐embedded material.

#### CINtec®PLUS (p16/Ki‐67 dual immunostaining)

Dual immunostaining with p16/Ki‐67 will be performed on a second ThinPrep® slide prepared using residual cytologic specimens, with the CINtec®PLUS Kit (Roche MTM Laboratories, Heidelberg, Germany) according to the manufacturer's instructions. Briefly, the anal cytology material will be stained at the University of Miami Pathology Laboratory using a mouse monoclonal antibody directed against human p16INKa (p16) (clone E6H4) and recombinant rabbit monoclonal antibody directed against human Ki‐67 (clone 274‐11AC3V1), using the BenchMark ULTRA instrument (Ventana Roche). Samples with insufficient cellularity will be reported as unsatisfactory and excluded from evaluation. A board‐certified cytopathologist will review all cases for the presence of positive cells demonstrating dual staining. A slide will be considered positive if one or more squamous epithelial cell(s) are stained positive for both p16 (brown cytoplasm) and Ki‐67 (red nuclei).

#### DNA methylation

DNA will be extracted from biopsy and swab samples using the DNeasy Blood and Tissue kit (Qiagen) and quantified using the Quant‐it PicoGreen Kit (Invitrogen). The EZ DNA Methylation Kit (Zymo Research, Orange, CA, USA) will be used to bisulfite‐convert 500 ng of DNA according to the manufacturer's instructions. Methylation analysis of *ASCL1, LHX8, ZNF582, SST, WDR17, ZIC1*, and *ST6GALNAC5* will be performed using standardized multiplex quantitative methylation‐specific PCR (qMSP) assays using the primers and methods previously described (van der Zee et al., 2021). Briefly, a ViiA 7 Real‐Time PCR System (Applied Biosystems, Foster City, CA, USA) will be used to conduct quantitative PCR to evaluate methylation of these markers relative to ß‐actin (ACTB), which will be used as a reference gene. Molecular biology grade water will be used as a negative control, and a DNA‐based calibrator (IDT) will be used as internal quality control. Cycle threshold (Cq) values will be measured for fluorescence at fixed thresholds for 32 cycles. ΔΔCq ratios will be computed by comparing the target Cq values to the Cq values of ACTB and the internal quality control calibrator (2 − ΔΔCq × 100) (Schmittgen & Livak, 2008).

#### Immune profiling

Anal samples collected by HRA‐guided biopsy at baseline, 1 year, and 2 years will undergo immunophenotyping by multicolor flow cytometry in Dr Velu's laboratory using the same immune profiling approach described in our previous studies (Govindaraj et al., 2024; Haddad et al., 2022). Approximately 1 × 10⁶ lymphocytes per sample will be stained with a panel of 34 extracellular and intracellular markers using fluorochrome‐conjugated antibodies. Details of all antibodies used for flow cytometry, including clone and manufacturer information, are provided in Table 1, encompassing markers for both innate and T‐cell immune populations in anal biopsy specimens. Dead cells will be excluded using a Live/Dead Fixable Near‐IR viability dye (Invitrogen, L10119). Cells will first be stained for surface markers, followed by fixation and permeabilization using Permeabilization Solution 2 for 10 min, and subsequent intracellular staining for FoxP3 and Ki‐67 for 30 min at room temperature. Cells stained with antibodies from the innate panel will not undergo subsequent intracellular antibody staining. Samples will then be washed with FACS buffer and centrifuged at 1500 rpm for 5 min. For short‐term storage (up to 16 h), cells will be fixed in 2% paraformaldehyde.

**Table 1:** Flow Cytometry Antibody Panel for T‐cell and Innate Immune Cell Profiling in Anal Biopsy Samples

Category	Marker	Clone	Company
Regulatory	FoxP3	206D	BioLegend
Exhaustion/activation	PD‐1	EH12.2H7	BioLegend
Exhaustion/activation	LAIR‐1	DX26	BD Biosciences
Activation/tissue resident	CD69	FN50	BioLegend
Activation	CD38	HIT2	BioLegend
Activation	HLA‐DR	G46‐6	BD Biosciences
Proliferation	Ki‐67	B56	BD Biosciences
Lymph node homing/memory	CCR7	G043H7	BioLegend
Tissue homing	CCR5	3A9	BD Biosciences
Tissue resident	CD103	2G5	Beckman Coulter
T cell	CD3	SP34‐2	BD Biosciences
T cell	CD4	L200	BD Biosciences
T cell	CD8	RPA‐T8	BD Biosciences
Pan‐leukocyte marker	CD45	H130	BD Biosciences
Differentiation	CD45RA	HI100	BD Biosciences
Antigen presentation (innate)	HLA‐DR	G46‐6	BD Biosciences
Innate activation	CD80	L307.4	BD Biosciences
Innate activation	CD86	2331	BD Biosciences
Myeloid‐derived suppressor	CD33	P67.6	BioLegend
Regulatory (innate)	PD‐L1	29E.2A3	BioLegend
Macrophage	CD163	GH1/61	BD Biosciences
Classical monocyte	CD14	M5E2	BD Biosciences
Myeloid dendritic cell	CD11c	3.9	BioLegend
Myeloid cells	CD11b	ICRF44	BD Biosciences
Plasmacytoid dendritic cell	CD123	9F5	BD Biosciences
Lymphoid lineage (innate panel)	CD3	SP34‐2	BD Biosciences
B cell	CD19	HIB19	BD Biosciences
NK cell	CD56	NACM16.2	BD Biosciences
Cytotoxic/Fc receptor	CD16	3G8	BioLegend
Neutrophils	CD66abc	TET2	Miltenyi Biotec
Pan‐leukocyte marker	CD45	H130	BD Biosciences
Viability	Live/Dead	‐	Invitrogen

Data acquisition will be performed on a BD LSRFortessa or BD FACSymphony flow cytometer (BD Biosciences, San Jose, CA, USA), with a minimum of 500,000 total events collected per sample. Data will be analyzed using FlowJo software (version X.0.7; Tree Star, Ashland, OR, USA). Analysis will be restricted to live cells, and all samples are expected to have >75% viability. Advanced analyses, including high‐dimensional visualization (e.g., viSNE and SPADE), may be performed using Cytobank. We have considerable experience in performing these assays in human tissues (Govindaraj et al., 2021). Collectively, these analyses will provide a comprehensive characterization of innate and T‐cell immune subsets and their functional states in biopsy specimens, their relationship to HPV infection, and the local anogenital mucosal immune microenvironment.

#### 10×single‐cell RNA sequencing

This will be used to identify the cellular component orchestrating the anal canal microenvironment. Single‐cell suspensions of 700–1200 cells/µL (min 50 µL) of anal biopsy samples from 20 individuals will be subjected to 10 × droplet capture within 4 h of collection. Cell viability of >90% will be recommended. The 10× controller chip using the Chromium Next GEM Single Cell 3’ kit (10X, PN‐1000121) will be used. The resulting 10× libraries will be sequenced using Illumina chemistry. Sequencing depth for 3’ and 5’ Gene Expression libraries will be >50,000 read pairs per cell. The Emory Integrated Genomics Core (EIGC) will perform the assay.

#### Peripheral blood collection and analysis

Approximately 8 mL of whole blood will be collected using BD Vacutainer CPT Sodium Citrate tubes (REF No. 362761), enabling standardized isolation of peripheral blood mononuclear cells (PBMCs). PBMCs from participants consenting to optional blood collection at Emory University will be used to profile innate immune markers and T‐cell–associated biomarkers via multicolor flow cytometry and single‐cell RNA sequencing. The use of CPT tubes ensures consistent PBMC separation across participants, supporting high‐quality and reproducible immunophenotyping (Chen et al., 2020).

#### Inflammatory protein markers

Anal swab concentrations (at baseline, Year 1, and Year 2) of inflammatory markers [IL1ra, IL1β, IL6, IL6sr, TNF‐α, sTNFR2, IL‐10, CRP, and interferon (IFN)‐γ] will be determined using multiplex assays (Meso Scale Discovery, Rockville, MD) according to the manufacturer's protocol. All samples will be run in duplicate and mean intra‐ and inter‐assay CVs will be calculated and expected to be <10%. These inflammatory markers have been reliably elevated in inflammation‐associated diseases, including anal inflammation and our data on head and neck cancer (van Onkelen et al., 2016; Xiao et al., 2018; Xiao et al., 2021).

### Criteria for discontinuing or modifying allocated interventions {11b}

Not applicable. No study‐assigned interventions will be administered.

### Strategies to improve adherence to interventions {11c}

Not applicable. No study‐assigned interventions will be administered.

### Relevant concomitant care permitted or prohibited during the trial {11d}

All participants will continue to receive standard clinical care throughout study participation. No restrictions will be placed on routine medical care, medications, or clinically indicated procedures.

### Provisions for post‐trial care {30}

Because ACCESS is an observational cohort study, no post‐trial intervention is planned. Participants diagnosed with aHSIL or anal cancer will receive appropriate clinical management and referrals according to current standard‐of‐care guidelines.

### Outcomes {12}

#### Primary outcome

The primary outcome is the detection of anal high‐grade aHSIL confirmed by histopathologic evaluation of HRA‐directed biopsies at baseline and follow‐up visits.

#### Secondary outcomes

1. Diagnostic performance of anal cytology, high‐risk HPV genotyping, and CINtec®PLUS dual staining for the detection of biopsy‐confirmed aHSIL, including sensitivity, specificity, positive predictive value, negative predictive value, and concordance measures
2. Association between anal DNA methylation markers (ZNF582, ASCL1, ZIC1, SST, LHX8, WDR17, and ST6GALNAC5) and aHSIL prevalence and clearance
3. Association between immune profiling markers, inflammatory biomarkers, and aHSIL prevalence and clearance
4. Characterization of cellular and molecular pathways associated with HPV infection and anal dysplasia using single‐cell RNA sequencing
5. Assessment of environmental, behavioral, socioeconomic, and clinical factors associated with aHSIL prevalence and clearance
6. Exploratory evaluation of concordance between self‐collected and provider‐collected anal swab specimens for high‐risk HPV detection and HPV genotyping

### Participant timeline {13}

Participants will be enrolled at baseline and followed annually for up to 2 years. Study procedures conducted at baseline, Year 1, and Year 2 include clinical assessments, questionnaires, DARE, HRA, anal cytology, HPV testing, CINtec®PLUS dual staining, DNA methylation analysis, inflammatory marker assessment, and collection of optional biospecimens. The allowable window for annual follow‐up visits is ±90 days from the target visit date. A summary of study procedures and participant assessments is provided in Table 2, and the overall study workflow is illustrated in Figure 1.

**Table 2:** Participant Timeline: Schedule of Study Procedures

Procedures	Baseline visit (Day 0)	Year 1 visit (±90 days)	Year 2 visit (±90 days)
Written informed consent	✓		
Eligibility confirmation/chart review	✓		
Demographic information	✓		
Medical history and clinical characteristics	✓	✓	✓
Environmental covariates questionnaires	✓	✓	✓
Behavioral and lifestyle questionnaires	✓	✓	✓
Charlson Comorbidity Index	✓	✓	✓
Digital anorectal examination	✓	✓	✓
High‐resolution anoscopy (HRA)	✓	✓	✓
HRA‐directed anal biopsy	✓	✓	✓
Anal cytology collection	✓	✓	✓
High‐risk HPV testing	✓	✓	✓
CINtec®PLUS Dual Stain	✓	✓	✓
DNA methylation analysis	✓	✓	✓
Inflammatory markers	✓	✓	✓
Optional self‐collected anal swab	✓	✓	✓
Optional additional biopsy for biorepository	✓	✓	✓
Optional blood collection (Emory only)	✓	✓	✓
Adverse event assessment	✓	✓	✓
Clinical management/referral if needed	✓	✓	✓

Annual follow‑up visit window: ±90 days from the baseline visit anniversary.

Anal biopsies are HRA‑directed and performed as part of standard clinical care; additional biopsy samples for biobanking are collected only with separate consent.

Blood collection is optional and performed at Emory University only.

Participants with aHSIL+ will receive treatment per standard clinical guidelines; participants with suspected cancer will be referred to oncology.

### Sample size {14}

A total of 500 participants will be enrolled, including 250 participants from Emory University and 250 participants from the University of Miami. Sample size calculations were based on the primary objective of evaluating the diagnostic performance of screening biomarkers for the detection of biopsy‐confirmed aHSIL. Assuming an aHSIL prevalence of 15% to 35% among high‐risk populations, power calculations demonstrated adequate power to assess sensitivity and specificity of screening markers and evaluate associations between molecular and immunologic biomarkers and aHSIL. Detailed power calculations are provided in Table 3.

**Table 3:** Power Calculation

Prevalence	Sensitivity power	Specificity power	Actual significance‐ sensitivity	Actual significance‐ specificity
0.15	0.4193	0.9556	0.0106	0.0111
0.20	0.5013	0.9351	0.0069	0.0105
0.25	0.6641	0.9251	0.0085	0.0115
0.30	0.7778	0.8915	0.0093	0.0107
0.35	0.8545	0.8745	0.0096	0.0097
0.40	0.9051	0.8544	0.0095	0.0105

### Recruitment {15}

Study participants will be recruited from Emory University clinical sites and from the University of Miami clinical sites. Though the study will be open to eligible individuals receiving care throughout both healthcare systems, most study participants will be recruited from the Grady Memorial Hospital Georgia Cancer Center of Excellence Colposcopy and Anoscopy Clinics and the Ponce de Leon Center Anoscopy Clinic at Emory University and from the Anal Dysplasia clinics at Jackson Memorial Hospital (JMH) and at the Sylvester Comprehensive Cancer Center (SCCC) at the University of Miami during routine scheduled anal cancer screening examinations. These clinics are supervised by Dr Flowers at Emory University and Dr Rosa‐Cunha at both sites for University of Miami. We will request a waiver of consent for screening potential participants for the study. Inclusion and exclusion criteria will be assessed by reviewing medical records and prescreening potential participants via the phone.

## Assignment of interventions: allocation

### Sequence generation {16a}

Not applicable. ACCESS is a prospective observational cohort study and does not involve randomization or allocation of participants to intervention groups.

### Concealment mechanism {16b}

Not applicable. No allocation sequence or intervention assignment will be used in this observational cohort study.

### Implementation {16c}

Not applicable. Participants will not be assigned to intervention groups. Eligible individuals will be enrolled consecutively based on study eligibility criteria.

## Assignment of interventions: Blinding

### Who will be blinded {17a}

Not applicable. The ACCESS study is a prospective observational cohort study and does not involve randomization, intervention allocation, or participant blinding.

### Procedure for unblinding if needed {17b}

Not applicable. No blinding procedures will be implemented because ACCESS is an observational cohort study and does not involve allocation of participants to intervention groups.

## Data collection and management

### Plans for assessment and collection of outcomes {18a}

Outcome, baseline, and follow‐up data will be collected using standardized questionnaires, electronic medical record review, and laboratory assessments. Demographic, clinical, behavioral, and environmental variables will be collected as described below.

#### Demographic, clinical, and environmental covariates

A standardized self‐reported questionnaire will collect demographic, clinical, and environmental information. The literacy level of the questionnaires will be at the 5th grade reading level; the questions are structured and minimize the use of free text responses. Questions will cover socioeconomic status, behavioral and lifestyle factors, and environmental context. Alcohol and tobacco use will be assessed based on questions from etiologic studies that have assessed these factors in relation to cancer risk. We will query both frequency of consumption and amount consumed by type of alcohol. “One drink” will refer to 12 ounces of beer, 4 ounces of wine, or 1.5 ounces of hard liquor. For tobacco use, we will assess smoking status; among current and former smokers, we will obtain information regarding pack‐years, as well as years since quitting if appropriate.

#### Sexual behaviors

The number of lifetime sexual partners will be defined as the total reported number of vaginal and anal sex partners. The lifetime number of anal sex partners and whether recent anal sex partners are new will also be collected. Self‐reported sexual orientation and sexual behaviors, such as heterosexual men/women or MSM, will be collected.

Clinical characteristics will be collected at baseline and followed up as appropriate, including history of aHSIL, treatment of aHSIL, anal carcinoma, abnormal anal cytology results, HPV vaccination, and other comorbidities. For participants living with HIV, the data collection will focus on time from HIV diagnosis, CD4 count, HIV viral load levels, major clinical events including hospitalizations and surgeries, antiretroviral history including types and duration, HIV‐associated conditions (opportunistic infections, neoplasms, etc.), and other concomitant medications. With the exception of comorbidities, which will be measured using the Charlson Comorbidity Index, all data will be collected at baseline and follow‐ups via electronic medical record review (Charlson et al., 1994).

We will use county health rankings (CHR), the area of deprivation index (ADI), and the residential segregation index to explore environmental factors. The environment is thought to have a key role in health outcomes and is included in WHO frameworks (WHO, 2010).

The CHR measures important health factors in communities across the US (Remington et al., 2015). The overall rankings in health factors are an estimate of the future health of counties as compared to other counties within a state. The ranks are based on health behaviors, clinical care, social and economic factors, and physical environment factors. Lower scores mean better health in a county.

The ADI reflects a geographic area's level of socioeconomic deprivation and is associated with health outcomes (Kind & Buckingham, 2018). The ADI accounts for income, education, employment, and housing quality at the neighborhood level, with lower scores indicating more affluent neighborhoods.

The residential segregation index is an index of dissimilarity using the nationwide American Community Survey as the data source, with higher values indicating greater segregation (CHR, 2025). Residential segregation has been linked to differences in socioeconomic mobility and neighborhood conditions across populations, which may influence health outcomes (CHR, 2025; Williams & Collins, 2001).

Laboratory assessments will include anal cytology, hrHPV genotyping, CINtec®PLUS dual staining, DNA methylation analysis, inflammatory marker assessment, immune profiling, and single‐cell RNA sequencing. Research staff will receive standardized training on participant assessment, biospecimen collection, and data entry procedures to promote data quality and consistency across study sites. Data will be captured and managed using REDCap and institutional databases.

### Plans to promote participant retention and complete follow‐up {18b}

Participant retention will be promoted through reminder emails, telephone calls, and text messages sent approximately 7 days, 3 days, and 1 day before scheduled study visits. Participants will receive compensation for completed study visits in accordance with approved study procedures. Follow‐up visits will be conducted within a ±90‐day window of the scheduled annual visit. Participants who discontinue study participation will be asked for permission to retain previously collected data and specimens, and available clinical outcome data may be obtained through medical record review when permitted by consent and institutional policies.

### Data management {19}

Study data will be collected and managed using REDCap electronic data capture tools hosted at Emory University and collaborating institutions. Data obtained from questionnaires, EMR review, laboratory assays, and study procedures will be entered into secure, password‐protected databases accessible only to authorized study personnel. Standardized data collection procedures and staff training will be implemented to promote data quality and consistency across study sites. Data validation checks, routine monitoring, and periodic quality assurance reviews will be conducted to identify and resolve data discrepancies. Participant identifiers will be stored separately from research data, and all analyses will be performed using de‐identified datasets. Study records and biological specimen information will be maintained in accordance with institutional policies, HIPAA requirements, and applicable regulatory guidelines.

### Confidentiality {27}

Personal information obtained from potential and enrolled participants will be collected and maintained in accordance with IRB, HIPAA, and institutional policies. Participant data will be stored in secure, password‐protected databases accessible only to authorized study personnel. Each participant will be assigned a unique study identification number, and identifying information will be stored separately from research data. Biological specimens and laboratory data will be labeled using study identifiers rather than personal information. Data shared between collaborating institutions will be de‐identified whenever possible and transferred through secure, approved mechanisms. All study records will be retained and protected according to institutional and regulatory requirements before, during, and after completion of the study.

### Plans for collection, laboratory evaluation, and storage of biological specimens for genetic or molecular analysis in future use {33}

Biological specimens collected as part of the ACCESS study will include anal swabs, HRA‐directed anal biopsy samples, and optional peripheral blood specimens obtained at baseline and follow‐up visits. Specimens will be processed according to standardized operating procedures and used for histologic evaluation, HPV genotyping, DNA methylation analysis, inflammatory marker assessment, immune profiling, and single‐cell RNA sequencing, as appropriate. Anal biopsy specimens will be processed for study‐related laboratory analyses and will not be stored for long‐term biobanking. PBMCs will be isolated from whole blood samples, suspended in cryopreservation medium, and stored for future analyses. Plasma aliquots will be stored at −80°C. All specimens will be labeled using unique study identification numbers and maintained in secure, monitored facilities accessible only to authorized study personnel. Participants will provide separate consent for future use of stored biological specimens in accordance with institutional, ethical, and regulatory requirements.

## Statistical methods

### Statistical methods for primary and secondary outcomes {20a}

All statistical analyses will be conducted using R and SAS software, along with established bioinformatics pipelines where appropriate. aHSIL will be classified as prevalent if detected at baseline (HRA biopsy‐confirmed) and as cleared if absent at follow‐up after treatment (Gillison et al., 2012; Muñoz et al., 2003; Wagner et al., 2019).

### Aim 1: Screening performance

The diagnostic performance of screening markers (CINtec ®PLUS, hrHPV, and anal cytology) for detecting aHSIL at baseline will be evaluated using sensitivity, specificity, positive predictive value (PPV), negative predictive value (NPV), and Cohen's kappa. Performance will be assessed for individual markers as well as combinations of markers (CINtec®PLUS+hrHPV, CINtec ®PLUS+anal cytology, hrHPV+anal cytology, and all three combined). Additionally, for all participants, we will use multivariable logistic regression to evaluate the association between the screening markers and aHSIL status at baseline, controlling for sex and important covariates. High‐risk group type (history of high‐grade anogenital tract neoplasia; solid organ transplant; HIV) will be included as a covariate. We will employ forward and backward stepwise selection regression to identify the best‐fit predictive model based on receiver operating characteristic (ROC) curves. The model with the highest area under the curve (AUC) will be chosen as the best predictive model for aHSIL at baseline (prevalence), similar to our previous work (under review). Covariates including socioeconomic status (race/ethnicity, marital status, education, and other socioeconomic factors), behavioral and lifestyle factors (alcohol drinking, tobacco smoking, sexual behaviors, etc.), and clinical characteristics will be considered. Bivariate correlations between each of the covariates and anal dysplasia will be examined. Covariates will be selected based on both statistical significance and clinical relevance. Clinically important covariates may be retained in the models, even if they do not meet the predefined significance threshold of p < 0.1.

### Aim 2: DNA methylation and immune profiling

#### Aim 2a: DNA methylation

Multivariable logistic regression models will be used to evaluate associations between individual DNA methylation markers (*ZNF582, ASCL1, ZIC1, SST, LHX8, WDR17*, and *ST6GALNAC5*) and aHSIL (prevalence and clearance). Separate models will be fitted for each marker, with aHSIL status as the outcome. Adjusted odds ratios (ORs) and 95% confidence intervals (CIs) will be reported. Baseline data will be used to evaluate prevalence, while longitudinal follow‑up data will be used to assess clearance. In these models, aHSIL status at baseline will be considered as the response, and each anal DNA methylation marker will be the predictor. Covariates will be selected in the same manner as in Aim 1. Model‐based ORs and 95% CIs will be reported, as well as predicted prevalence. For the clearance analysis, only participants treated for aHSIL will be used for modeling. Clearance will be defined as no detectable aHSIL at the 1‐year follow‐up visit (for those with baseline aHSIL) or the 2‐year follow‐up visit (for those with newly diagnosed aHSIL cases at the 1‐year visit), or any follow‐up visit after aHSIL treatment. Similar logistic regression models as those for prevalence will be used for clearance analysis to assess association between clearance and methylation markers (at the same point at the same time). In addition, a secondary analysis using similar logistic regression models will investigate the prognostic ability of methylation markers at aHSIL diagnosis (prior to treatment) with subsequent 1‐year clearance. Stratified analyses will repeat the above analysis for each of the three high‐risk groups. For primary (prevalence) analysis, methylation markers will be assessed at α = 0.006 using a Bonferroni adjustment to account for multiple testing.

#### Aim 2b: Immune profiling and inflammatory markers

Associations between immune cell populations, inflammatory markers, and aHSIL (prevalence and clearance) will be assessed using multivariable logistic regression models. Separate models will be fitted for each immune and inflammatory marker.

In addition to flow cytometry, we will perform 10× single‐cell RNA sequencing on the anal biopsy samples for a total of 40 participants using a 2 × 2 design. We will include participants with high‐risk HPV‐16 persistent infection (N = 20) versus participants with no detectable HPV in at least two consecutive visits (N = 20). Each of the groups will also include individuals with aHSIL+ (N = 10) versus <aHSIL (N = 10). We will demultiplex raw base call data into FASTQ files and a count matrix; we will then identify and remove low‐quality or empty cells. The R package Seurat will be used for all downstream analyses (Butler et al., 2018). The count data will be filtered and log transformed. A principal component analysis of the most variable genes will be performed, and an elbow plot will be used to select the principal components (PCs) capturing the most variance in the dataset. These PCs will be used as edge weights in an unsupervised graph‐based clustering to identify cell clusters. T‐distributed stochastic neighbor embedding (tSNE) will be used for visualization of the cell clusters between groups. Expression levels of cell‐type‐specific markers will be used to determine the putative identities of each cell cluster. Once the cell clusters are identified, differential expression will be assessed using one of the four tests in Seurat. We define differentially expressed genes as those with an adjusted p‐valuee ≤ 0.05 and an average log2 fold change ≥ 1. We will also perform pathway enrichment analysis on the enriched population from each group and compare them for their functional, inflammatory, and immunosenescent pathways using IPA between the groups.

#### Power calculation

For Aim 1, based on our previous work and literature, we expect 15%–35% prevalence of aHSIL at baseline for the three high‐risk groups. Power estimates for sensitivity and specificity across a range of prevalence values are summarized in Table 3.

Using results from a meta‐analysis of anal cytology as a screening tool, we assume an anal cytology sensitivity of 0.7 and a specificity of 0.59. As we plan to compare four combinations of the screening markers to the anal cytology referent, we adjust for multiple testing by setting significance at 0.0125. Primary analysis will focus on the entire cohort at baseline (n = 500 participants). A total sample size of n = 500 achieves the sensitivity power described in Table 3 using a two‐sided binomial test comparing sensitivities of 0.7 and 0.83 under the null and alternative hypotheses, respectively. This sample size also achieves the specificity power listed in Table 3 using a two‐sided binomial test comparing specificities of 0.59 and 0.68 under the null and alternative hypotheses, respectively. For Aim 2, we plan to compare 7 methylation markers and 35 cellular markers. We therefore adjust for multiple testing by setting the significance level at 0.006 for each independent variable of interest. A logistic regression of a binary response variable (aHSIL status) on a continuous, normally distributed variable (X; methylation marker, immunological/inflammatory marker) with a sample size of n = 500 participants achieves 81% power at a 0.006 significance level to detect an odds ratio of 1.5 when the prevalence of aHSIL (Y) in the population is 0.3. This odds ratio is the ratio of the odds that Y = 1 when the independent variable (X) is one standard deviation above its mean to the odds that Y = 1 when X is equal to its mean. Power is calculated in PASS 2024.

### Interim analyses {21b}

No formal interim analyses or stopping guidelines are planned. Study progress, data quality, participant enrollment, and retention will be monitored throughout the study by the investigative team.

### Methods for additional analyses (e.g., subgroup analyses) {20b}

Additional analyses will include stratified and subgroup analyses by high‐risk group, sex, immune status, HPV status, and aHSIL status, as appropriate.

### Analysis methods to handle protocol non‐adherence and any statistical methods to handle missing data {20c}

Because ACCESS is an observational cohort study, participants who discontinue follow‐up or miss study visits will remain included in analyses when data are available. Missing data patterns will be evaluated, and appropriate statistical approaches, including complete‐case analyses and multiple imputation methods when warranted, may be applied. Sensitivity analyses will be conducted as appropriate to assess the impact of missing data on study findings.

### Plans to give access to the full protocol, participant‐level data, and statistical code {31c}

The study protocol will be publicly available through publication. De‐identified participant‐level data and statistical code may be made available upon reasonable request to the corresponding investigators and in accordance with institutional policies, ethical approvals, participant consent, and NIH data‐sharing requirements.

## Oversight and monitoring

### Composition of the coordinating center and trial steering committee {5d}

The ACCESS study is coordinated jointly by Emory University and the University of Miami. The coordinating team is responsible for study oversight, participant recruitment, protocol implementation, data collection, quality assurance, regulatory compliance, and study management across participating sites. The principal investigators and key study personnel meet regularly to review enrollment, protocol adherence, data quality, biospecimen collection, and study progress. Because ACCESS is a prospective observational cohort study, a formal Trial Steering Committee has not been established.

### Composition of the data monitoring committee, its role, and reporting structure {21a}

A formal Data Monitoring Committee (DMC) has not been established because ACCESS is a prospective observational cohort study involving minimal risk procedures and no investigational interventions. Study oversight will be provided by the principal investigators and study leadership at Emory University and the University of Miami. Study progress, participant enrollment, protocol compliance, biospecimen collection, and data quality will be reviewed regularly by the investigative team.

#### Adverse event reporting and harms {22}

Although ACCESS is an observational cohort study, adverse events related to study procedures, including blood collection, anal swab collection, HRA, and biopsy procedures, will be documented and reviewed. Any adverse events reported by participants or identified by study personnel will be assessed and managed according to institutional policies and reported to the appropriate IRBs and regulatory bodies as required. Participants requiring medical evaluation or treatment will be referred to their healthcare providers and managed according to standard clinical practice.

#### Frequency and plans for auditing trial conduct {23}

The ACCESS study will be conducted in accordance with the approved protocol, IRB requirements, and applicable institutional policies. Study records, regulatory documentation, and data quality procedures may be reviewed periodically by institutional oversight bodies and authorized regulatory personnel, as required. No formal independent auditing committee is planned due to the observational nature of the study.

### Plans for communicating important protocol amendments to relevant parties (e.g., trial participants, ethical committees) {25}

Any significant protocol modifications, including changes to study procedures, eligibility criteria, outcomes, or analyses, will be submitted for review and approval by the appropriate IRBs prior to implementation. Approved amendments will be communicated to study investigators, research staff, and participants when applicable. Revisions affecting participant consent or study participation will be incorporated into updated consent documents and communicated according to institutional and regulatory requirements.

### Dissemination plans {31a}

Results from the ACCESS study will be disseminated through presentations at scientific meetings, publications in peer‐reviewed journals, and presentations to healthcare professionals and relevant stakeholder groups. Study findings may also be shared with participants upon request and through institutional communication channels when appropriate. De‐identified aggregate results will be reported in accordance with NIH and institutional policies. There are no publication restrictions imposed by the study funders. All publications and presentations arising from the study will be reviewed and approved by the study investigators prior to dissemination.

## Discussion

Results from this study will be interpreted within the framework of anal carcinogenesis, which is driven by viral persistence, host immune responses, and molecular alterations.

In this study, we anticipate that integration of molecular biomarkers, including HPV genotyping, DNA methylation profiles, and immune signatures, will improve identification of aHSIL compared to cytology alone. We further expect that biomarker‐based stratification will distinguish lesions with a higher likelihood of persistence and progression from those that regress.

Consistent with Aim 1, this study will evaluate the diagnostic performance of novel biomarkers, including CINtec®PLUS and high‐risk HPV testing, compared with traditional anal cytology for the detection of HRA biopsy–proven aHSIL. We expect these approaches, particularly when used in combination, to improve screening accuracy and reduce unnecessary high‐resolution anoscopies.

In Aim 2, concurrent immune profiling is expected to clarify the role of the local inflammatory and immune microenvironment in lesion behavior, while epigenetic alterations may serve as indicators of lesion persistence and progression potential. In parallel, we will investigate the underlying biology of aHSIL by integrating anal DNA methylation markers with immune profiling, including assessments of innate and adaptive immunity as well as inflammatory states, using both tissue and exfoliative samples.

Together, these multiomics data will enable a more refined understanding of anal carcinogenesis as a dynamic interaction between viral persistence, host immunity, and environmental influences. Overall, the integration of screening biomarkers, molecular signatures, and immune profiling is expected to provide a more comprehensive understanding of aHSIL biology. These findings may support the development of more precise, risk‐stratified screening and prevention strategies in high‐risk populations.

A potential limitation of this study is that participant recruitment is conducted through existing health care systems, which may underrepresent individuals with limited health care access. To address this, the study includes comprehensive assessment of social determinants of health, including economic deprivation, housing instability, food insecurity, health care access, and neighborhood‐level disadvantage, to better understand their relationship with HPV‐associated disease outcomes and anal cancer risk. Collectively, the strengths of this study include its prospective design, multi‐institutional recruitment, focus on diverse high‐risk populations, and integration of clinical, molecular, immunological, and environmental data. We expect the findings to improve screening accuracy, elucidate mechanisms of lesion persistence versus regression, and ultimately inform more precise prevention strategies. This work represents an important step toward reducing anal cancer disparities and advancing precision prevention in vulnerable populations.

### Trial status

Trials guidance: Protocol version 1.0, August 2026.

The ACCESS study is an ongoing prospective, multisite observational cohort study. The funded project period extends from September 12, 2023, and is anticipated to be completed by August 2028. Participants will be followed annually for up to 2 years following enrollment.

### Author Contributions {31b}

Canhua Xiao, Lisa Flowers, Isabella Rosa‐Cuhna, and Vijayakumar Velu conceived and designed the study. Nadi N. Kaonga, Mary Grace Tadros, Patricia Paule Jeudin, Kadera Jana Lymon, Nichole A. Ciers‐Davis, Annabel Reyes, Sakthivel Govindaraj, Monica T. Garcia‐Buitrago, C. Christina Mehta, Johnny H. Galli, JoNell E. Potter, Michaela Peters, Alicia K. Smith, Chris Ibegbu, and Marina Mosanjac collected data and provided critical feedback on protocol development. Raju Subbiah, Sohan Rodney Bangera, and Vijayakumar Velu wrote the main manuscript text. All authors reviewed, edited, and approved the final version of the manuscript. Sohan Rodney Bangera and Raju Subbiah contributed equally to this work and are considered co‐first authors. All authors read and approved of the final manuscript.

### Funding {4}

This study is supported by the National Institutes of Health (NIH) and the National Cancer Institute (NCI) through award R01CA285198. VV's lab is supported by the Emory National Biomedical Research Center through NIH grant no. ORIP/OD P51OD011132. Funding supports participant recruitment, biospecimen collection, laboratory analyses, data management, statistical analysis, and study coordination activities at participating institutions. The funding agency had no role in the design of the study; collection, management, analysis, or interpretation of data; preparation of the manuscript; or the decision to submit the protocol for publication. Final authority over all study activities and publication decisions resides with the study investigators.

### Competing Interests {28}

The authors declare that they have no competing interests.

### Availability of Data and Materials {29}

The final privacy‐protected study dataset will be available to the study investigators and authorized research personnel. Data access will be managed in accordance with participant consent, institutional policies, and applicable regulatory requirements. De‐identified data may be shared with qualified researchers upon reasonable request and appropriate approvals. There are no contractual restrictions limiting investigator access to the final dataset.

### Ethics Approval and Consent to Participate {24}

The study was reviewed and approved by the Emory University Institutional Review Board (IRB# BIO 01302023) and the Grady Health System Research Oversight Committee. Written informed consent will be obtained from all participants prior to enrollment and before any study‐related procedures are performed.

### Consent for Publication {32}

Not applicable. This manuscript does not contain any individual participant data, images, videos, or other identifiable information requiring consent for publication.

## References

[cpz170518-bib-0001] Alemany, L. , Saunier, M. , Alvarado‐Cabrero, I. , Quirós, B. , Salmeron, J. , Shin, H. R. , Pirog, E. C. , Guimerà, N. , Hernández‐Suárez, G. , & Félix, A. (2015). Human papillomavirus DNA prevalence and type distribution in anal carcinomas worldwide. International Journal of Cancer, 136(1), 98–107. 10.1002/ijc.28963 PMC4270372 24817381

[cpz170518-bib-0002] Bojko, M. M. , Kucejko, R. J. , & Poggio, J. L. (2018). Racial disparities and the effect of county level income on the incidence and survival of young men with anal cancer. Health Equity, 2(1). 10.1089/heq.2018.0018 PMC6110184 30283867

[cpz170518-bib-0003] Butler, A. , Hoffman, P. , Smibert, P. , Papalexi, E. , & Satija, R. (2018). Integrating single‐cell transcriptomic data across different conditions, technologies, and species. Nature Biotechnology, 36(5), 411–420. 10.1038/nbt.4096 PMC6700744 29608179

[cpz170518-bib-0004] Charlson, M. , Szatrowski, T. P. , Peterson, J. , & Gold, J. (1994). Validation of a combined comorbidity index. Journal of Clinical Epidemiology, 47(11), 1245–1251. 10.1016/0895-4356(94)90129-5 7722560

[cpz170518-bib-0005] Chen, H. , Schürch, C. M. , Noble, K. , Kim, K. , Krutzik, P. O. , O'Donnell, E. , Vander Tuig, J. , Nolan, G. P. , & McIlwain, D. R. (2020). Functional comparison of PBMCs isolated by cell preparation tubes (CPT) vs. lymphoprep tubes. BMC Immunology, 21(1), 15. 10.1186/s12865-020-00345-0 PMC7106580 32228458

[cpz170518-bib-0006] Darragh, T. M. , Colgan, T. J. , Cox, J. T. , Heller, D. S. , Henry, M. R. , Luff, R. D. , McCalmont, T. , Nayar, R. , Palefsky, J. M. , & Stoler, M. H. (2012). The lower anogenital squamous terminology standardization project for HPV‐associated lesions: Background and consensus recommendations from the College of American Pathologists and the American Society for Colposcopy and Cervical Pathology. Archives of Pathology & Laboratory Medicine, 136(10), 1266–1297. 10.5858/arpa.LGT200570 22742517

[cpz170518-bib-0007] Dean, H. D. , & Fenton, K. A. (2010). Addressing social determinants of health in the prevention and control of HIV/AIDS, viral hepatitis, sexually transmitted infections, and tuberculosis. Public Health Reports, 125(4_suppl), 1–5. 10.1177/00333549101250S40 PMC2882967 20629250

[cpz170518-bib-0008] Deshmukh, A. A. , Suk, R. , Shiels, M. S. , Sonawane, K. , Nyitray, A. G. , Liu, Y. , Gaisa, M. M. , Palefsky, J. M. , & Sigel, K. (2020). Recent trends in squamous cell carcinoma of the anus incidence and mortality in the United States, 2001–2015. JNCI: Journal of the National Cancer Institute, 112(8), 829–838. 10.1093/jnci/djz219 PMC7825484 31742639

[cpz170518-bib-0009] Gaisa, M. , Sigel, K. , Hand, J. , & Goldstone, S. (2014). High rates of anal dysplasia in HIV‐infected men who have sex with men, women, and heterosexual men. Aids, 28(2), 215–222. 10.1097/QAD.0000000000000062 24072194

[cpz170518-bib-0010] Gillison, M. L. , Broutian, T. , Pickard, R. K. , Tong, Z.‐y. , Xiao, W. , Kahle, L. , Graubard, B. I. , & Chaturvedi, A. K. (2012). Prevalence of oral HPV infection in the United States, 2009‐2010. JAMA, 307(7), 693–703. 10.1001/jama.2012.101 PMC5790188 22282321

[cpz170518-bib-0011] Govindaraj, S. , Babu, H. , Endress, D. , Rahman, S. J. , Ibegbu, C. , Kozlowski, D. S. , Doraivelu, K. , Herring, G. B. , Mehta, C. , & Smith, A. K. (2021). Accumulation of tissue resident NK cells in female genital tract during follicular phase of HIV seronegative women. The Journal of Immunology, 206(1_Supplement), 55.04–55.04. 10.4049/jimmunol.206.Supp.55.04

[cpz170518-bib-0012] Govindaraj, S. , Tyree, S. , Herring, G. B. , Rahman, S. J. , Babu, H. , Ibegbu, C. , Young, M. R. , Mehta, C. , Haddad, L. B. , & Smith, A. K. (2024). Differential expression of HIV target cells CCR5 and α4β7 in tissue resident memory CD4 T cells in endocervix during the menstrual cycle of HIV seronegative women. Frontiers in Immunology, 15, 1456652. 10.3389/fimmu.2024.1456652 PMC11461385 39386203

[cpz170518-bib-0013] Haddad, L. B. , Herring, G. B. , Mehta, C. C. , Staple, T. , Young, M. R. , Govindaraj, S. , Velu, V. , & Smith, A. K. (2022). Evaluating the impact of three progestin‐based hormonal contraceptive methods on immunologic changes in the female genital tract and systemically (CHIME Study): A prospective cohort study protocol. BMC Women's Health, 22(1), 456. 10.1186/s12905-022-02053-w PMC9673204 36401326

[cpz170518-bib-0014] Heard, I. , Etienney, I. , Potard, V. , Poizot‐Martin, I. , Moore, C. , Lesage, A.‐C. , Ressiot, E. , Crenn‐Hebert, C. , Fléjou, J.‐F. , & Cubie, H. (2015). High prevalence of anal human papillomavirus–associated cancer precursors in a contemporary cohort of asymptomatic HIV‐infected women. Clinical Infectious Diseases, 60(10), 1559–1568. 10.1093/cid/civ049 25645213

[cpz170518-bib-0015] Herd, P. , Palloni, A. , Rey, F. , & Dowd, J. B. (2018). Social and population health science approaches to understand the human microbiome. Nature Human Behaviour, 2(11), 808–815. 10.1038/s41562-018-0452-y PMC6711373 31457107

[cpz170518-bib-0016] Hernandez, J. M. , Siegel, E. M. , Riggs, B. , Eschrich, S. , Elahi, A. , Qu, X. , Ajidahun, A. , Berglund, A. , Coppola, D. , & Grady, W. M. (2012). DNA methylation profiling across the spectrum of HPV‐associated anal squamous neoplasia. PloS One, 7(11), e50533. 10.1371/journal.pone.0050533 PMC3511539 23226306

[cpz170518-bib-0017] Kind, A. J. , & Buckingham, W. R. (2018). Making neighborhood‐disadvantage metrics accessible—the neighborhood atlas. The New England Journal of Medicine, 378(26), 2456. 10.1056/NEJMp1802313 PMC6051533 29949490

[cpz170518-bib-0018] Korn, A. R. , Walsh‐Bailey, C. , Pilar, M. , Sandler, B. , Bhattacharjee, P. , Moore, W. T. , Brownson, R. C. , Emmons, K. M. , & Oh, A. Y. (2022). Social determinants of health and cancer screening implementation and outcomes in the USA: A systematic review protocol. Systematic Reviews, 11(1), 117. 10.1186/s13643-022-01995-4 PMC9175338 35676720

[cpz170518-bib-0019] Lee, G. C. , Kunitake, H. , Milch, H. , Savitt, L. R. , Stafford, C. E. , Bordeianou, L. G. , Francone, T. D. , & Ricciardi, R. (2018). What is the risk of anal carcinoma in patients with anal intraepithelial neoplasia III? Diseases of the Colon & Rectum, 61(12), 1350–1356. 10.1097/DCR.0000000000001219 PMC6219933 30303884

[cpz170518-bib-0020] Liu, Y. , Gaisa, M. M. , Wang, X. , Swartz, T. H. , Arens, Y. , Dresser, K. A. , Sigel, C. , & Sigel, K. (2018). Differences in the immune microenvironment of anal cancer precursors by HIV status and association with ablation outcomes. The Journal of Infectious Diseases, 217(5), 703–709. 10.1093/infdis/jix454 PMC5853940 28968881

[cpz170518-bib-0021] Lorincz, A. T. , Nathan, M. , Reuter, C. , Warman, R. , Thaha, M. A. , Sheaff, M. , Vasiljevic, N. , Ahmad, A. , Cuzick, J. , & Sasieni, P. (2017). Methylation of HPV and a tumor suppressor gene reveals anal cancer and precursor lesions. Oncotarget, 8(31), 50510. 10.18632/oncotarget.17984 PMC5584159 28881579

[cpz170518-bib-0022] Machalek, D. A. , Poynten, M. , Jin, F. , Fairley, C. K. , Farnsworth, A. , Garland, S. M. , Hillman, R. J. , Petoumenos, K. , Roberts, J. , & Tabrizi, S. N. (2012). Anal human papillomavirus infection and associated neoplastic lesions in men who have sex with men: A systematic review and meta‐analysis. The Lancet Oncology, 13(5), 487–500. 10.1016/S1470-2045(12)70080-3 22445259

[cpz170518-bib-0023] Maness, S. B. , & Thompson, E. L. (2019). Social determinants of human papillomavirus vaccine uptake: An assessment of publicly available data. Public Health Reports, 134(3), 264–273. 10.1177/0033354919838219 PMC6505323 30951641

[cpz170518-bib-0024] Muñoz, N. , Bosch, F. X. , de Sanjosé, S. , Herrero, R. , Castellsagué, X. , Shah, K. V. , Snijders, P. J. , & Meijer, C. J. (2003). Epidemiologic classification of human papillomavirus types associated with cervical cancer. New England Journal of Medicine, 348(6), 518–527. https://doi.rog/10.1056/NEJMoa021641 10.1056/NEJMoa021641 12571259

[cpz170518-bib-0025] Palefsky, J. M. , Lee, J. Y. , Jay, N. , Goldstone, S. E. , Darragh, T. M. , Dunlevy, H. A. , Rosa‐Cunha, I. , Arons, A. , Pugliese, J. C. , & Vena, D. (2022). Treatment of anal high‐grade squamous intraepithelial lesions to prevent anal cancer. New England Journal of Medicine, 386(24), 2273–2282. 10.1056/NEJMoa2201048 PMC9717677 35704479

[cpz170518-bib-0026] Remington, P. L. , Catlin, B. B. , & Gennuso, K. P. (2015). The county health rankings: Rationale and methods. Population Health Metrics, 13(1), 11. 10.1186/s12963-015-0044-2 PMC4415342 25931988

[cpz170518-bib-0027] Roberts, J. R. , Siekas, L. L. , & Kaz, A. M. (2017). Anal intraepithelial neoplasia: A review of diagnosis and management. World Journal of Gastrointestinal Oncology, 9(2), 50–61. 10.4251/wjgo.v9.i2.50 PMC5314201 28255426

[cpz170518-bib-0028] Roberts, J. R. , Siekas, L. L. , & Kaz, A. M. Anal intraepithelial neoplasia: A review of diagnosis and management. World Journal of Gastrointestinal Oncology, 9(2), 50–61. 10.4251/wjgo.v9.i2.50 PMC5314201 28255426

[cpz170518-bib-0029] Schmittgen, T. D. , & Livak, K. J. (2008). Analyzing real‐time PCR data by the comparative CT method. Nature Protocols, 3(6), 1101–1108. 10.1038/nprot.2008.73 18546601

[cpz170518-bib-0030] Stenzel, A. E. , Schlecht, N. F. , & Moysich, K. B. (2021). Racial/ethnic disparities in survival among women diagnosed with invasive cancer of the anal canal: An analysis of surveillance, epidemiology, and end results (SEER) data. Journal of Gastrointestinal Cancer, 52(3), 854–862. 10.1007/s12029-020-00472-2 32803517

[cpz170518-bib-0031] Stewart, D. B. , Gaertner, W. B. , Glasgow, S. C. , Herzig, D. O. , Feingold, D. , & Steele, S. R. (2018). The American Society of Colon and Rectal Surgeons clinical practice guidelines for anal squamous cell cancers (revised 2018). Diseases of the Colon & Rectum, 61(7), 755–774. 10.1097/DCR.0000000000001114 29878949

[cpz170518-bib-0032] Tinmouth, J. , Peeva, V. , Amare, H. , Blitz, S. , Raboud, J. , Sano, M. , Steele, L. , & Salit, I. E. (2016). Progression from perianal high‐grade anal intraepithelial neoplasia to anal cancer in HIV‐positive men who have sex with men. Diseases of the Colon & Rectum, 59(9), 836–842. 10.1097/DCR.0000000000000644 27505112

[cpz170518-bib-0033] van der Zee, R. P. , Richel, O. , van Noesel, C. J. , Ciocănea‐Teodorescu, I. , van Splunter, A. P. , Ter Braak, T. J. , Nathan, M. , Cuming, T. , Sheaff, M. , & Kreuter, A. (2021). Cancer risk stratification of anal intraepithelial neoplasia in human immunodeficiency virus–positive men by validated methylation markers associated with progression to cancer. Clinical Infectious Diseases, 72(12), 2154–2163. 10.1093/cid/ciaa397 PMC8204787 32266940

[cpz170518-bib-0034] van der Zee, R. P. , Richel, O. , van Noesel, C. J. , Novianti, P. W. , Ciocanea‐Teodorescu, I. , van Splunter, A. P. , Duin, S. , van den Berk, G. E. , Meijer, C. J. , & Quint, W. G. (2019). Host cell deoxyribonucleic acid methylation markers for the detection of high‐grade anal intraepithelial neoplasia and anal cancer. Clinical Infectious Diseases, 68(7), 1110–1117. 10.1093/cid/ciy601 PMC6424081 30060049

[cpz170518-bib-0035] van Onkelen, R. , Gosselink, M. P. , van Meurs, M. , Melief, M.‐J. , Schouten, W. R. , & Laman, J. (2016). Pro‐inflammatory cytokines in cryptoglandular anal fistulas. Techniques in Coloproctology, 20(9), 619–625. 10.1007/s10151-016-1494-7 PMC5003909 27402195

[cpz170518-bib-0036] Wagner, S. , Roberson, D. , Boland, J. , Yeager, M. , Cullen, M. , Mirabello, L. , Dunn, S. T. , Walker, J. , Zuna, R. , & Schiffman, M. (2019). Development of the TypeSeq assay for detection of 51 human papillomavirus genotypes by next‐generation sequencing. Journal of Clinical Microbiology, 57(5). 10.1128/jcm.01794-18 PMC6498001 30814267

[cpz170518-bib-0037] Walsh, T. , Bertozzi‐Villa, C. , & Schneider, J. A. (2015). Systematic review of racial disparities in human papillomavirus–associated anal dysplasia and anal cancer among men who have sex with men. American Journal of Public Health, 105(4), e34–e45. 10.2105/AJPH.2014.302469 PMC4358202 25713941

[cpz170518-bib-0038] Wentzensen, N. , Follansbee, S. , Borgonovo, S. , Tokugawa, D. , Sahasrabuddhe, V. V. , Chen, J. , Lorey, T. S. , Gage, J. C. , Fetterman, B. , & Boyle, S. (2014). Analytic and clinical performance of cobas HPV testing in anal specimens from HIV‐positive men who have sex with men. Journal of Clinical Microbiology, 52(8), 2892–2897. 10.1128/jcm.03517-13 PMC4136172 24899025

[cpz170518-bib-0039] Williams, D. R. , & Collins, C. (2001). Racial residential segregation: A fundamental cause of racial disparities in health. Public Health Reports, 116(5), 404. 10.1093/phr/116.5.404 PMC1497358 12042604

[cpz170518-bib-0040] Xiao, C. , Beitler, J. J. , Higgins, K. A. , Glazer, T. , Huynh, L. K. , Paul, S. , Felger, J. C. , Wommack, E. C. , Saba, N. F. , & Shin, D. M. (2018). Associations among human papillomavirus, inflammation, and fatigue in patients with head and neck cancer. Cancer, 124(15), 3163–3170. 10.1002/cncr.31537 PMC6097898 29742284

[cpz170518-bib-0041] Xiao, C. , Beitler, J. J. , Peng, G. , Levine, M. E. , Conneely, K. N. , Zhao, H. , Felger, J. C. , Wommack, E. C. , Chico, C. , & Jeon, S. (2021). Epigenetic age acceleration, fatigue, and inflammation in patients undergoing radiation therapy for head and neck cancer: A longitudinal study. Cancer, 127(18), 3361–3371. 10.1002/cncr.33641 34027995

[cpz170518-bib-0042] Yaghoobi, M. , le Gouvello, S. , Aloulou, N. , Duprez‐Dutreuil, C. , Walker, F. , & Sobhani, I. (2011). FoxP3 overexpression and CD1a+ and CD3+ depletion in anal tissue as possible mechanisms for increased risk of human papillomavirus‐related anal carcinoma in HIV infection. Colorectal Disease, 13(7), 768–773. 10.1111/j.1463-1318.2010.02283.x 20394639

[cpz170518-bib-0043] Zhang, J. , Martins, C. R. , Fansler, Z. B. , Roemer, K. L. , Kincaid, E. A. , Gustafson, K. S. , Heitjan, D. F. , & Clark, D. P. (2005). DNA methylation in anal intraepithelial lesions and anal squamous cell carcinoma. Clinical Cancer Research, 11(18), 6544–6549. 10.1158/1078-0432.CCR-05-0374 16166431

[cpz170518-bib-0045] https://www.countyhealthrankings.org/health‐data/community‐conditions/social‐and‐economic‐factors/safety‐and‐social‐support/residential‐segregation‐blackwhite?year=2025

[cpz170518-bib-0046] *C.H.R. (2025). CHR. Residential Segregation ‐ Black/White*.

[cpz170518-bib-0047] https://seer.cancer.gov/statfacts/html/anus.html

[cpz170518-bib-0048] *SEER, N. C. I. (2026). SEER Cancer Stat Facts: Anal Cancer. Surveillance, Epidemiology, and End Results Program. Surveillance, Epidemiology, and End Results (SEER) Program, National Cancer Institute*.

[cpz170518-bib-0049] https://www.who.int/publications/i/item/9789241500852

[cpz170518-bib-0050] *WHO. (2010). A Conceptual Framework for Action on the Social Determinants of Health 13 July 2010| Report. Retrieved April 3^rd^, 2026*.

